# Supplementary material for: T cell receptor β-chain-targeting chimeric antigen receptor T cells against T cell malignancies
Source: Nat Commun. 2022 Jul 26;13:4334. doi: 10.1038/s41467-022-32092-8 (PMC9325690; doi:10.1038/s41467-022-32092-8)
Supplement: Supplementary file 1 — Supplementary Information [file 41467_2022_32092_MOESM1_ESM.pdf]

Supplementary Information

T cell receptor β-chain-targeting chimeric antigen receptor T cells against T cell malignancies

Fanlin Li†, Huihui Zhang†, Wanting Wang, Puyuan Yang, Yue Huang, Junshi Zhang, Yaping Yan, Yuan Wang, Xizhong Ding，Jie Liang, Xinyue Qi, Min Li, Ping Han, Xiaoqing Zhang, Xin Wang, Jiang Cao, Yang-Xin Fu and Xuanming Yang*

Supplemental Data for Figures:

Supplementary Fig. 1, related to Figure 1.
Supplementary Fig. 2, related to Figure 1.
Supplementary Fig. 3, related to Figure 1.
Supplementary Fig. 4, related to Figure 1.
Supplementary Fig. 5, related to Figure 3.
Supplementary Fig. 6, related to Figure 3.
Supplementary Fig. 7, related to Figure 5.
Supplementary Fig. 8, related to Figure 6.
Supplementary Fig. 9, related to Figure 6.
Supplementary Fig. 10, related to Figure 6.
Supplementary Fig. 11, related to Figure 6.

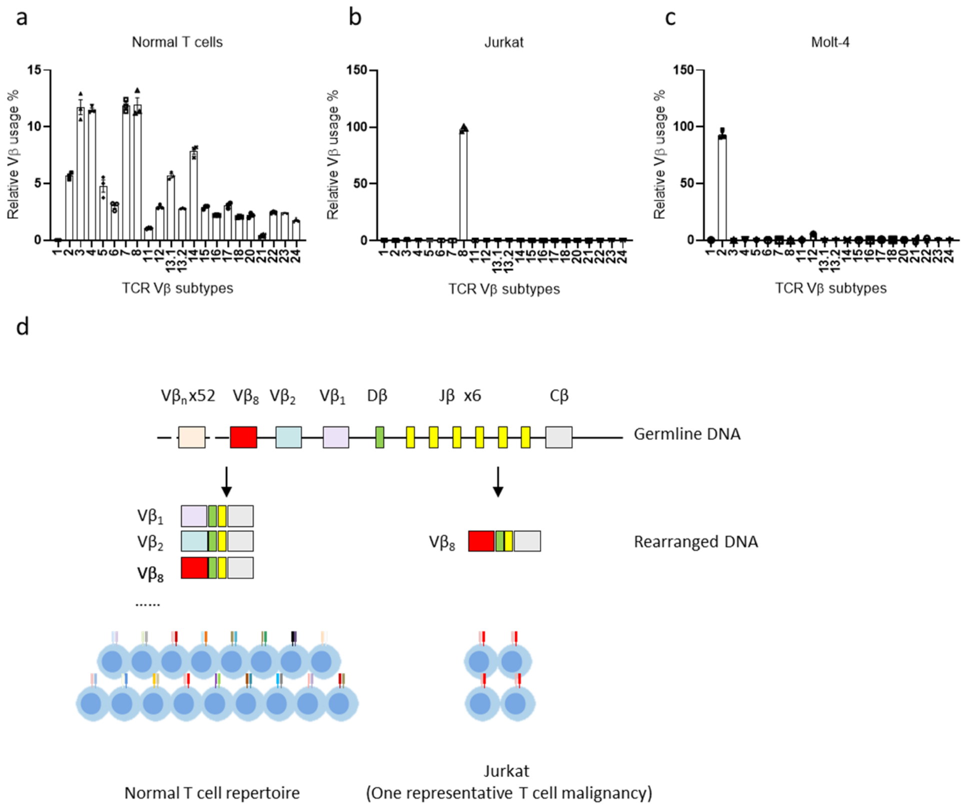

Supplementary Figure 1. TCR Vβ usage in normal and malignant T cells. **a-c** Real-time quantitative PCR analysis of TCR Vβ mRNA-expression levels (n = 3 biologically independent samples/group) in normal T cells from PBMCs (**a**), Jurkat T cell leukemia cells (**b**) and Molt-4 cells (**c**). **d** Schematic representation of different TCR Vβ usage in normal T cell repertoire and malignancy. The data shown represented the mean ± SEM. Representative results of one from three replicate experiments were shown in panels **a-c**. The cartoons in this figure were created with BioRender.com.

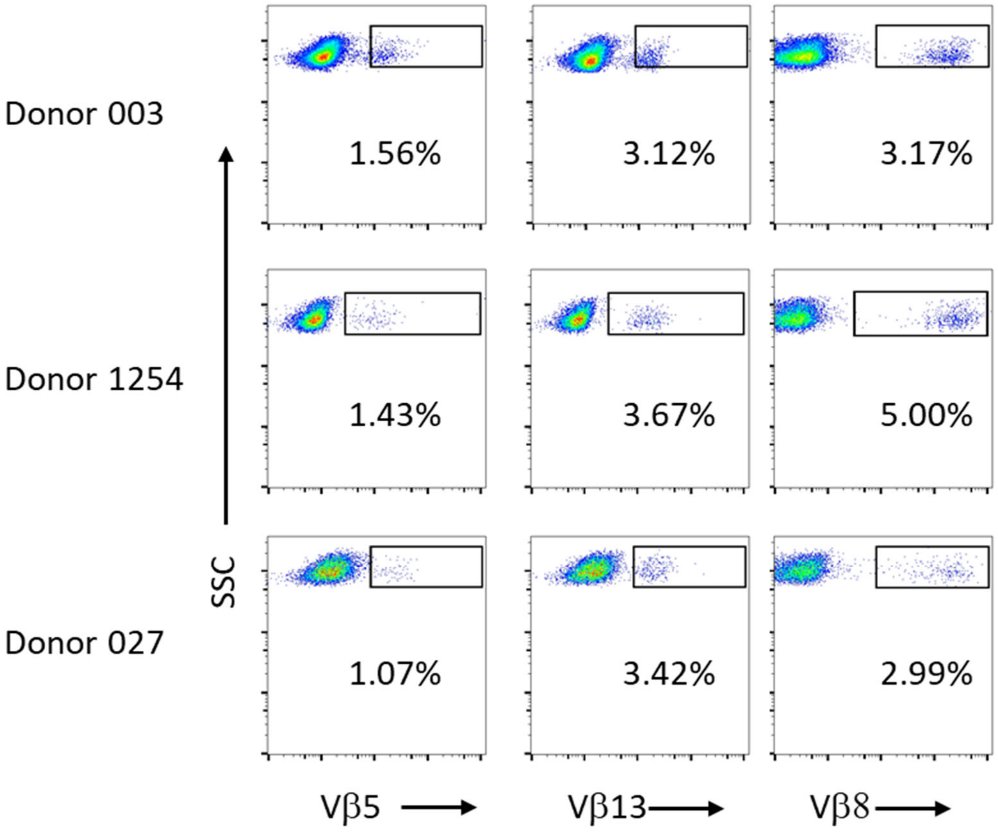

Supplementary Figure 2. TCR Vβ specific antibody recognized a small portion of normal T cells. Normal T cells of peripheral blood of indicated donors were stained with commercially available anti-Vβ5, Vβ13, or Vβ8 antibody and analyzed by flow cytometry.

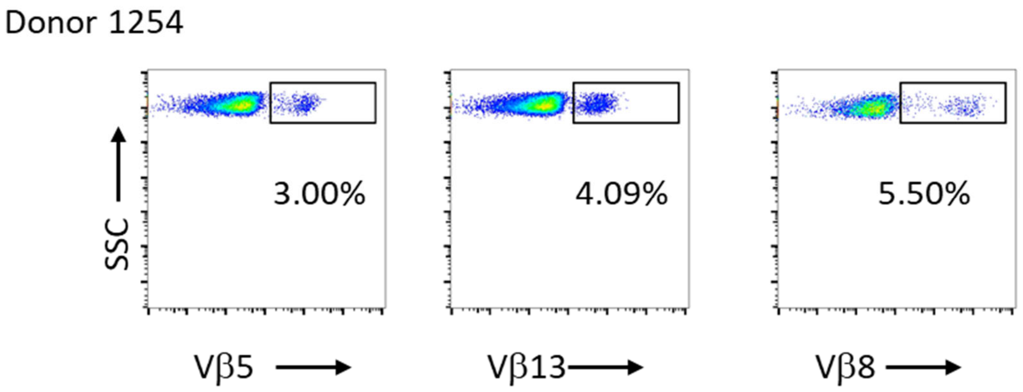

Supplementary Figure 3. Home-made recombinant TCR Vβ specific antibody recognized a small portion of normal T cells. Normal T cells of peripheral blood of indicated donor were stained with home-made recombinant anti- Vβ5, Vβ13, or Vβ8 antibody and analyzed by flow cytometry.

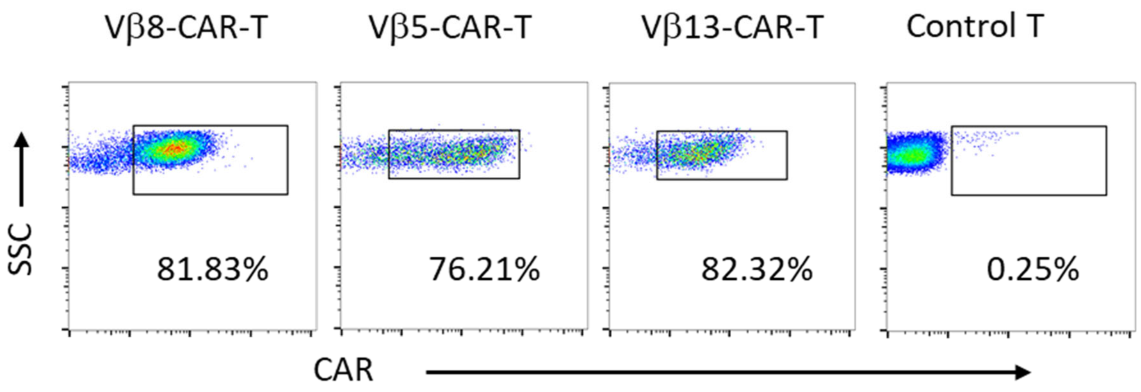

Supplementary Figure 4. CAR expression of different TCR Vβ specific CAR-T cells. Mock T cells or indicated CAR-T cells were stained with anti-mouse Fab antibody and analyzed by flow cytometry.

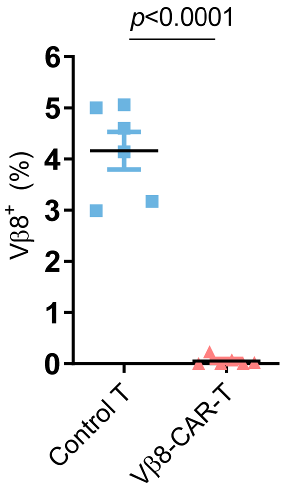

Supplementary Figure 5. Vβ8 expression in different donor-derived CAR-T cells. Control T cells and Vβ8-CAR-T cells from donor #228, #1254, #Z003, #108, #024 and #027 were stained with anti-TCR Vβ8 antibody and analyzed by flow cytometry (n = 6 biologically independent samples/group). The data shown represented the mean ± SEM. Statistical significances were determined by two-sided unpaired t-tests. The normality of the data was confirmed by Shapiro–Wilk test.

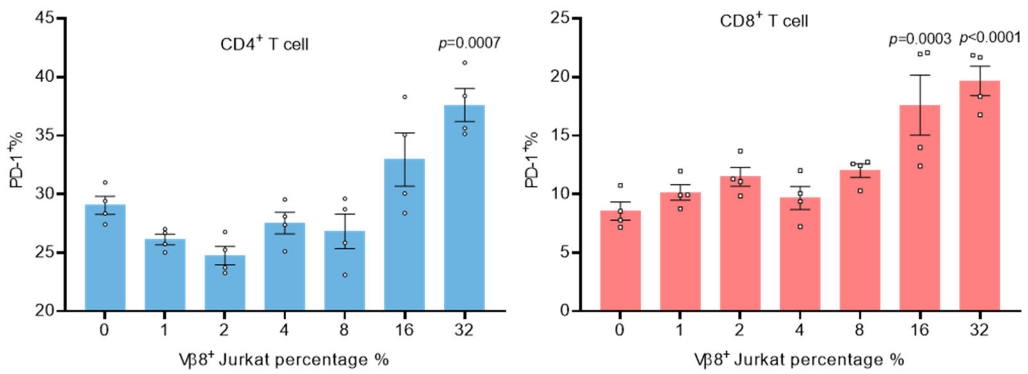

Supplementary Figure 6. CAR-T cell exhaustion analysis. Vβ8-CAR-T cells were cultured with indicated percentage of Jurkat cells. The expression of PD-1 were analyzed by flow cytometry (n = 4 biologically independent samples/group). The data shown represented the mean ± SEM. Statistical significances were determined by two-sided, one-way ANOVA with Dunnett's multiple comparisons correction.

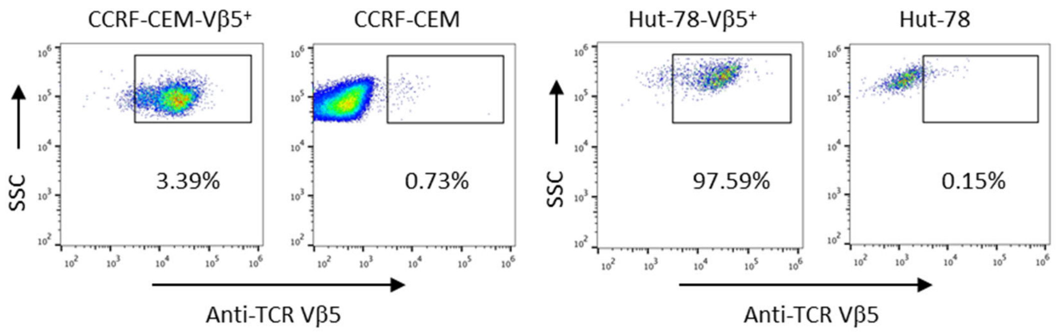

Supplementary Figure 7. Construction of TCR Vβ5 expressing CCRF-CEM and Hut-78 cells. Parental and TCR Vβ5 expressed CCRF-CEM and Hut-78 cells were stained with anti-TCR Vβ5 antibody and analyzed by flow cytometry.

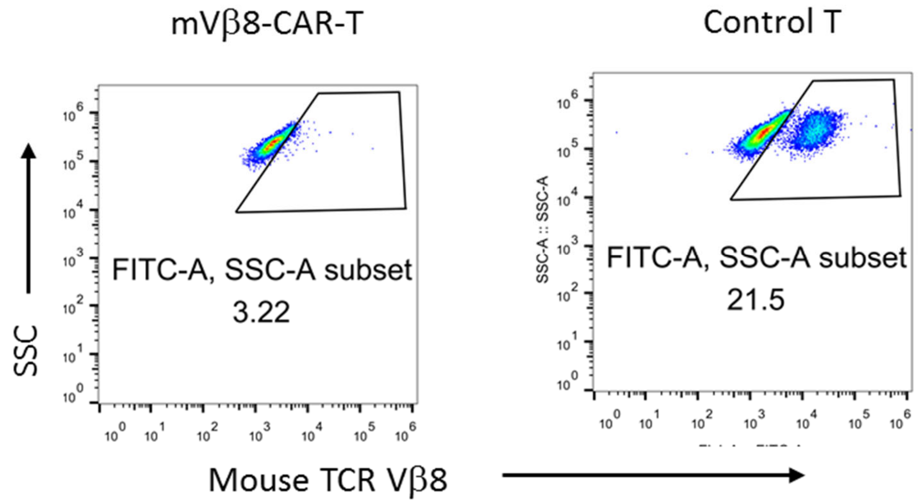

Supplementary Figure 8. Mouse Vβ8-CAR-T cells specifically targeted Vβ8^{+} primary mouse T cells. Control T cells or mVβ8-CAR-T cells were stained with anti-mouse Vβ8 antibody and analyzed by flow cytometry.

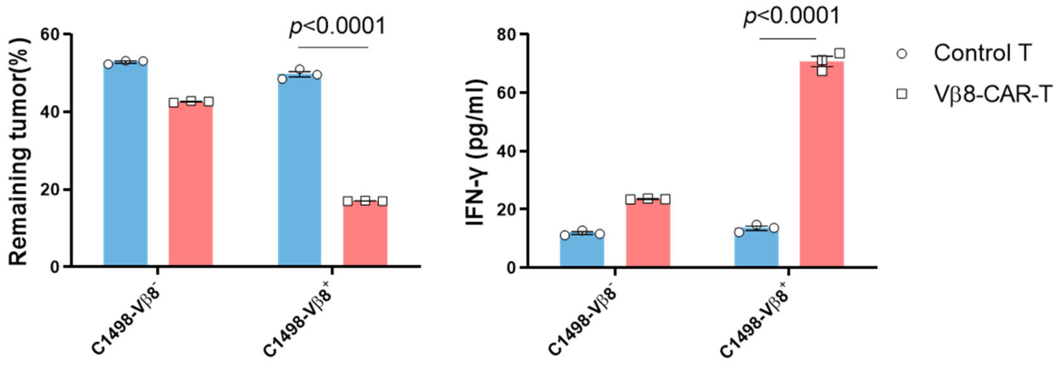

Supplementary Figure 9. Mouse Vβ8-CAR-T cells specifically killed Vβ8$^+$ tumor cells. Untransduced T cells or Vβ8-CAR-T cells were co-cultured with C1498-Vβ8$^-$ or C1498-Vβ8$^+$ cells in triplicate wells at a CAR-T: C1498 cell ratio of 2:1 for 24 h (n = 3 biologically independent samples/group). The indicated cytokines in the supernatants were analyzed by CBA assay. The data shown represented the mean ± SEM. Statistical significances were determined by two-sided, two-way ANOVA with sidaks multiple comparisons correction.

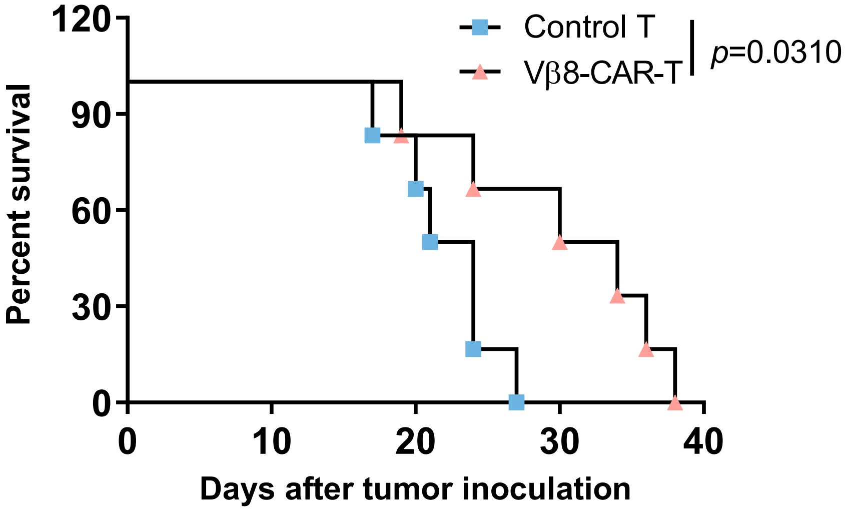

Supplementary Figure 10. Mouse Vβ8-CAR-T cells prolonged survival of Vβ8$^+$ tumor bearing mice (n = 6 mice/group). NSG mice were intravenously inoculated with 2.5 × 10^5 C1498-Vβ8$^+$ tumor cells. Four days later, tumor-bearing mice were treated with 3 × 10^6 un-transduced or mouse Vβ8-CAR-T cells. Kaplan–Meier OS curves were shown for the inoculated mice. Statistical significances were determined by the two-sided Mantel–Cox test. Representative results of one from two replicate experiments were shown.

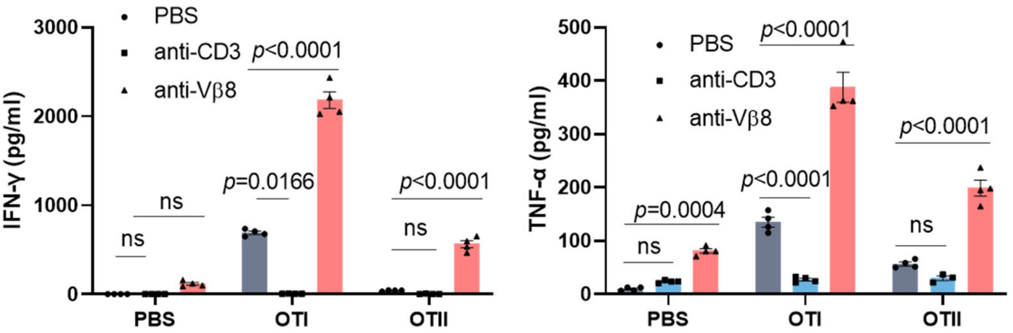

Supplementary Figure 11. T cell-mediated immunity after TCR Vβ8$^+$ cell depletion. WT B6 mice were intraperitoneally injected with 200 μg of anti-CD3 or anti-TCR Vβ8 antibodies, or PBS on days -2 and 5. The mice were vaccinated with 10 μg of the DEC-OVA fusion protein and 100 μg of an anti-CD40 antibody as an adjuvant on day 0. On day 12, splenocytes were stimulated with OT-I or OT-II peptides. Two days later, indicated cytokines ware analyzed by CBA assay (n = 4 biologically independent samples/group). The data shown represented the mean ± SEM. Statistical significances were determined by two-sided, one-way ANOVA with Dunnett's multiple comparisons corrections. ns, not significant. Representative results of one from two replicate experiments were shown.
