# Supplementary material for: T cell receptor β-chain-targeting chimeric antigen receptor T cells against T cell malignancies
Source: Nat Commun. 2022 Jul 26;13:4334. doi: 10.1038/s41467-022-32092-8 (PMC9325690; doi:10.1038/s41467-022-32092-8)
Supplement: Supplementary file 3 — Reporting Summary [file 41467_2022_32092_MOESM3_ESM.pdf]

nature portfolio

Corresponding author(s): Xuanming Yang

Last updated by author(s): 05/15/2022

Reporting Summary

Nature Portfolio wishes to improve the reproducibility of the work that we publish. This form provides structure for consistency and transparency in reporting. For further information on Nature Portfolio policies, see our Editorial Policies and the Editorial Policy Checklist.

Statistics

For all statistical analyses, confirm that the following items are present in the figure legend, table legend, main text, or Methods section.

n/a	Confirmed	
☐	☒	The exact sample size (*n*) for each experimental group/condition, given as a discrete number and unit of measurement
☐	☒	A statement on whether measurements were taken from distinct samples or whether the same sample was measured repeatedly
☐	☒	The statistical test(s) used AND whether they are one- or two-sided<br>*Only common tests should be described solely by name; describe more complex techniques in the Methods section.*
☐	☒	A description of all covariates tested
☐	☒	A description of any assumptions or corrections, such as tests of normality and adjustment for multiple comparisons
☐	☒	A full description of the statistical parameters including central tendency (e.g. means) or other basic estimates (e.g. regression coefficient) AND variation (e.g. standard deviation) or associated estimates of uncertainty (e.g. confidence intervals)
☐	☒	For null hypothesis testing, the test statistic (e.g. *F*, *t*, *r*) with confidence intervals, effect sizes, degrees of freedom and *P* value noted<br>*Give P values as exact values whenever suitable.*
☒	☐	For Bayesian analysis, information on the choice of priors and Markov chain Monte Carlo settings
☒	☐	For hierarchical and complex designs, identification of the appropriate level for tests and full reporting of outcomes
☒	☐	Estimates of effect sizes (e.g. Cohen's *d*, Pearson's *r*), indicating how they were calculated

Our web collection on statistics for biologists contains articles on many of the points above.

Software and code

Policy information about availability of computer code

Data collection	Flow cytometry data were collected on CytoFLEX S (Beckman Coulter) with CyoExpert software V2.3 (Beckman Coulter) .<br>qRT-PCR data were collected on qTOWER3G touch (Analytik Jena AG) with qPCRsoft software V4.0 (Analytik Jena AG).
Data analysis	Flow cytometry data were analyzed with Flowjo V10 (BD).<br>qRT-PCR data were analyzed with qPCRsoft software V4.0 (Analytik Jena AG).<br>All the statistical analyses were performed on GraphPad Prism v8.0 (GraphPad Software).

For manuscripts utilizing custom algorithms or software that are central to the research but not yet described in published literature, software must be made available to editors and reviewers. We strongly encourage code deposition in a community repository (e.g. GitHub). See the Nature Portfolio guidelines for submitting code & software for further information.

Data

Policy information about availability of data

All manuscripts must include a data availability statement. This statement should provide the following information, where applicable:

- Accession codes, unique identifiers, or web links for publicly available datasets
- A description of any restrictions on data availability
- For clinical datasets or third party data, please ensure that the statement adheres to our policy

All data are available in the main text or the Supplementary Materials.

Field-specific reporting

Please select the one below that is the best fit for your research. If you are not sure, read the appropriate sections before making your selection.

☒ Life sciences ☐ Behavioural & social sciences ☐ Ecological, evolutionary & environmental sciences

For a reference copy of the document with all sections, see nature.com/documents/nr-reporting-summary-flat.pdf

Life sciences study design

All studies must disclose on these points even when the disclosure is negative.

Sample size	The sample sizes were chosen based on previous experience of similar experiments to produce statistically difference among different groups. For in vitro killing and T cell activation assay, 3 -4 biological samples were chosen for each group. For in vivo tumor models, 5-7 mice were chosen for each group.
Data exclusions	No data were excluded.
Replication	All in vitro studies and in vivo animal studies were repeated 3 or more times under similar experimental conditions. All attempts at replication were successful.
Randomization	For tumor experiments, mice were allocated to different groups with similar tumor burden. For in vitro killing and T cell activation assay, cells were randomly assigned to the different treatment groups.
Blinding	The investigators were not blinded to group allocation during data collection and analysis. We did not consider blinding of animal studies because the information of the moue strain and interventions is necessary for accurately conducting the experiments and for safety of researchers and the supporting team of the animal facility. The researchers were not blinded for in vitro experiments to practically conduct the experiments.

Reporting for specific materials, systems and methods

We require information from authors about some types of materials, experimental systems and methods used in many studies. Here, indicate whether each material, system or method listed is relevant to your study. If you are not sure if a list item applies to your research, read the appropriate section before selecting a response.

Materials & experimental systems

n/a	Involved in the study	
☐	☒	Antibodies
☐	☒	Eukaryotic cell lines
☒	☐	Palaeontology and archaeology
☐	☒	Animals and other organisms
☒	☐	Human research participants
☒	☐	Clinical data
☒	☐	Dual use research of concern

Methods

n/a	Involved in the study	
☒	☐	ChIP-seq
☐	☒	Flow cytometry
☒	☐	MRI-based neuroimaging

Antibodies

Antibodies used	Goat anti-mouse IgG, F(ab')2 fragment specific (115-605-006, 1:2500, Jackson ImmunoResearch), anti-human Fc (109-005-098, 1:2500, Jackson ImmunoResearch), anti-mouse Fab(115-005-071, 1:2500, Jackson ImmunoResearch) human Vb8 (348104, clone JR2, 1:250, BioLegend), Vb5 (349301, clone MH3-2, 1:250, BioLegend), Vb13 (362402, clone H131, 1:250, BioLegend), active caspase-3 (560626, clone C92-605, 1:250, BD Biosciences), granzyme B (372206, clone QA16A02, 1:250, BioLegend), mouse CD45 (103126, clone 30-F11, 1:250, BioLegend), TCRVb8.1 (118405, clone KJ16-133.18, 1:250, BioLegend), TCRb (109205, clone H57-597, 1:250, BioLegend), and Kb-SIINFEKL-pentamer (F093-2A-PE) (1:50, PROIMMUNE), rat anti-mouse CD40 (FGK45) (BE0016-2).
Validation	These antibodies are validated by the provider and have passed the routine QC test by the manufacturer.<br>Alexa Fluor 647 AffiniPure Goat Anti-Mouse IgG, F(ab')$_2$ fragment specific (115-605-006, 1:2500): https://www.jacksonimmuno.com/catalog/products/115-605-006.<br>AffiniPure Goat Anti-Human IgG, Fcγ fragment specific (109-005-098, 1:2500): https://www.jacksonimmuno.com/catalog/products/109-005-098.<br>AffiniPure Goat Anti-Mouse IgG, Fcγ fragment specific (115-005-071, 1:2500): https://www.jacksonimmuno.com/catalog/products/115-005-071.<br>Rabbit Alexa Fluor 647 Rabbit Anti-Active Caspase-3(560626, 1:250): https://www.bdbiosciences.com/zh-cn/products/reagents/flow-cytometry-reagents/research-reagents/single-color-antibodies-ruo/alexa-fluor-647-rabbit-anti-active-caspase-3.560626.<br>Mouse FITC anti-human/mouse Granzyme B Recombinant Antibody (372206, 1:250): https://www.biolegend.com/en-us/products/

fitc-anti-human-mouse-granzyme-b-recombinant-antibody-14430.
Rat Pacific Blue anti-mouse CD45 antibody (103126,1:250): https://www.biolegend.com/en-us/products/pacific-blue-anti-mouse-cd45-antibody-3102
Mouse PE anti-human CD4 Antibody (317410, 1:250): https://www.biolegend.com/en-us/products/pe-anti-human-cd4-antibody-3654
Mouse APC/Fire 750 anti-human CD8a Antibody (300932, 1:250): https://www.biolegend.com/en-us/products/apcfire-750-anti-human-cd8a-antibody-17305.
Mouse APC-eFluor 780 Monoclonal Antibody PD-1 (47-4714-82, 1:250): https://www.thermofisher.cn/cn/zh/antibody/product/CD279-PD-1-Antibody-clone-eBioJ105-J105-Monoclonal/47-2799-42.
Mouse PE anti-human TCR Vβ8 Antibody (348104， 1:250): https://www.biolegend.com/en-us/products/pe-anti-human-tcr-vbeta8-antibody-6502
Mouse Purified anti-human TCR Vβ5 Antibody (349301,1:250): https://www.biolegend.com/en-us/products/purified-anti-human-tcr-vbeta5-antibody-6742
Mouse Purified anti-human TCR Vβ13.1 Antibody (362402,1:250): https://www.biolegend.com/en-us/products/purified-anti-human-tcr-vbeta13-1-antibody-9774
Rat FITC anti-mouse TCR Vβ8.1, 8.2 antibody (118405,1:250): https://www.biolegend.com/en-us/products/fitc-anti-mouse-tcr-vbeta8-1-8-2-antibody-4651
Armenian Hamster FITC anti-mouse TCR β chain Antibody (109205,1:250): https://www.biolegend.com/en-us/products/fitc-anti-mouse-tcr-beta-chain-antibody-270
Kb-SIINFEKL-pentamer(F093-2A-PE) (1:50, PROIMMUNE): https://www.proimmune.com/ecommerce/pdf_files/Kb_SIINFEKL.pdf
Rat anti-mouse CD40 (FGK45) (BE0016-2): https://bxcell.com/product/m-cd40/
Rat anti-mouse CD16/CD32 antibody (2.4G2)(BE0307): https://bxcell.com/product/invivomab-anti-mouse-cd16-cd32/
Human TruStain FcX™ (Fc Receptor Blocking Solution) (422302): https://www.biolegend.com/en-us/products/human-trustain-fcx-fc-receptor-blocking-solution-6462

Eukaryotic cell lines

Policy information about cell lines

Cell line source(s)	The Lenti-X 293 cell line was purchased from Clontech. The Phoenix-ECO cell line was purchased from ATCC. Jurkat, Molt-4, CCRF-CEM and Hut-78 cells were kindly provided by Stem Cell Bank (Chinese Academy of Sciences). C1498 cells were kindly provided by Dr. Justin Kline (University of Chicago) and originally from ATCC. B16-OVA cells were kindly provided by Dr. Hans Schreiber (University of Chicago) and not available from commercial source.
Authentication	None of the cell lines used were authenticated.
Mycoplasma contamination	All cell lines are tested negative for mycoplasma.
Commonly misidentified lines (See ICLAC register)	No commonly misidentified lines were used in this study.

Animals and other organisms

Policy information about studies involving animals; ARRIVE guidelines recommended for reporting animal research

Laboratory animals	NOD-PrkdcscidIL2rytm1 mice (#NM-NSG-001) were purchased from Shanghai Model Organisms Center, Inc. C57BL/6 mice (# 213) were obtained from Beijing Vital River Laboratory Animal Technology Co., Ltd (Beijing, China).<br>Age from 6 week to 8 weeks.<br>Gender: female mice were used.<br>The mice were housed with a dark/light cycle of 12 hours, humidity of 40-70% and ambient temperature of 20-26C.
Wild animals	No wild animals were used in this study.
Field-collected samples	No field-collected samples were used in this study.
Ethics oversight	All mice were maintained under SPF conditions. Animal care and use were in accordance with institutional and NIH protocols and guidelines, and all studies were approved by the Animal Care and Use Committee of Shanghai Jiao Tong University.

Note that full information on the approval of the study protocol must also be provided in the manuscript.

Flow Cytometry

Plots

Confirm that:

☒ The axis labels state the marker and fluorochrome used (e.g. CD4-FITC).

☒ The axis scales are clearly visible. Include numbers along axes only for bottom left plot of group (a 'group' is an analysis of identical markers).

☒ All plots are contour plots with outliers or pseudocolor plots.

☒ A numerical value for number of cells or percentage (with statistics) is provided.

Methodology

Sample preparation	For cell-surface staining, cells were incubated for 10 min in staining buffer (1× phosphate-buffered saline [PBS] with 1% FBS) with antibodies recognizing CD16 and CD32 (anti-FcγRII and Fc?RIII, clone 2.4 G2), or Human TruStain FcX (BioLegend) to block nonspecific Fc-mediated binding. The blocked samples were subsequently stained with conjugated antibodies. Intracellular staining was performed following fixation for 30 min at room temperature in 4% paraformaldehyde and permeabilization with 1× Perm/Wash buffer (eBioscience) for 60 min at 4C. The cells were incubated with the indicated antibodies diluted in 1× Perm/Wash buffer for 30 min at 4C. The cells were analyzed using a CytoFLEX S flow cytometer (Beckman Coulter), and the data were analyzed with FlowJo software (Becton Dickinson).
Instrument	Cytoflex S (Beckman Coulter)
Software	Flowjo v10 (BD)
Cell population abundance	Cell population abundance was not analyzed in this study.
Gating strategy	Cells were firstly gated based on FSC/SSC. Then 7AAD- cells were gated as live cells for further analysis.

☒ Tick this box to confirm that a figure exemplifying the gating strategy is provided in the Supplementary Information.
